# Delirium Knowledge, Risk Factors, and Attitude Among the General Public in Saudi Arabia: A Cross-Sectional Study

**DOI:** 10.7759/cureus.59263

**Published:** 2024-04-29

**Authors:** Kareema Alshurtan, Fatimah Ali Alshammari, Alhanouf B Alshammari, Shatha H Alreheili, Shadan Aljassar, Jassam A Alessa, Hisham A Al Yateem, Manahel Almutairi, Alanud F Altamimi, Hamad A Altisan

**Affiliations:** 1 Internal Medicine, University of Hail College of Medicine, Hail, SAU; 2 College of Medicine, University of Hail, Hail, SAU; 3 Medicine and Surgery, University of Hail College of Medicine, Hail, SAU

**Keywords:** awareness, attitudes, knowledge, kingdom of saudi arabia, nurses, cognitive assessment, public awareness, risk factors, delirium

## Abstract

Introduction

Delirium is a common and serious neuropsychiatric disorder, of acute onset, present at any age, but more common in older adults, and very common in clinical practice. It combines mental and behavioral symptoms with a fluctuating course, with worsening of the condition in the afternoon and at night, with important repercussions on increased mortality, greater risk of cognitive impairment, and hospitalization costs. Delirium's impact extends to patients, families, and healthcare systems, emphasizing the need for public awareness and education in Saudi Arabia.

Methodology

It is a cross-sectional conducted in Saudi Arabia that aims to assess knowledge, risk factors, and attitudes regarding delirium among all Saudi and non-Saudi residents aged 18 and older. A 36 self-administered questionnaire, standardized Nordic, was used. Data were cleaned in Microsft Excel (Microsoft Corporation, USA) and analyzed using IBM SPSS Statistics (IBM Corp., Armonk, NY). This study was conducted in Saudi Arabia from May 2023 till March 2024.

Results

Our study involved 1,470 participants from Saudi Arabia, primarily females (79.1%), Saudi nationals (89.9%), and unmarried individuals (65.4%). Most participants were aged 18-24 (59.5%) and held bachelor's degrees (57.3%). Commonly recognized delirium risk factors included increasing age (63.3%), dementia (58.2%), and longer ICU stays (48.7%). The participants showed moderate knowledge of delirium symptoms and consequences. Attitudes varied, with many agreeing that delirium requires intervention (30.7%) but fewer considering it preventable (17.1%). Sociodemographic factors, including gender and age, significantly influenced knowledge and attitudes, while education levels did not.

Conclusion

Our study found that gender and age influenced knowledge and attitudes, highlighting the importance of targeted education. Future research should further investigate the effectiveness of such interventions in enhancing knowledge and awareness and promoting preventive actions.

## Introduction

Delirium is defined as a disturbance of consciousness that is characterized by acute onset with a fluctuating course of impaired awareness and attention with one or more additional disturbances in cognitive function [1], which is due to a wide range of factors, including medical conditions, severe neuropsychiatric diseases such as depression, and dementia among the elderly population, substances, medicine abuse, or combined cause [2]. Delirium can alternate in duration and severity, and it can be a temporary stage that is overcome by removing the causative etiologies or factors. Moreover, there are several risk factors, including the presence of three to five comorbidities, nonemployment, and illiteracy. Even so, there are some cases of unrecoverable delirium, which becomes a chronic cognitive disorder.

Although delirium can occur at any age, older individuals are at the highest risk for developing the condition with an advanced medical condition or cognitive dysfunction, which can lead to undesirable outcomes, including increased risk of falls, rapid decline in cognitive functions, prolonged length of hospital stay, increased risk of mortality and morbidity, and increased healthcare utilization and costs [3,4].

According to the fifth edition of the Diagnostic and Statistical Manual of Mental Diseases (DSM-V), combined with symptoms of delirium, scholars have divided delirium into four different clinical subtypes hyperactive and hypoactive delirium, mixed delirium, and no motor delirium [3]. The most common subtype of delirium is hypoactive delirium, which is characterized by confusion, sedation, apathy, unresponsiveness, motor delay, attitudinal withdrawal, and drowsiness [5,6]. Relatively poor prognoses, stressful injuries, and higher case fatality rates are most likely to occur in patients with hypoactive delirium [7,8]. Anxiety, agitation, and the removal of medical devices (e.g., intravenous catheters, masks, drainage tubes, and catheters) are characteristics of hyperactive delirium. Studies show that hyperactive delirium, in comparison to other subtypes, had a higher rate of unplanned extubation, particularly nasogastric and endotracheal intubation [9]. The prevalence of hyperactivity delirium is roughly 15% [10,11]. Mixed delirium is alternating symptoms of hypoactivity and hyperactivity with a prevalence rate ranging up to 54.9% in some studies [10,11,12]. Mechanical ventilation time, ICU days, overall delirium duration, and total hospital stay time of mixed delirium are more prolonged than those of other subtypes [7,11,13]. No motor subtype of delirium is represented by an altered state of consciousness without psychomotor disturbance, such as hyperkinesia or bradykinesia [9,14,15,16].

Delirium can be underdiagnosed or difficult to diagnose substantially due to the lack of routine cognitive assessment in hospitals and the fluctuation in signs and symptoms; hence, it can be present with pre-existing neurocognitive dysfunction, such as pre-existing dementia, which is one of the most critical risk factors, with studies showing that at least two-thirds of all delirium cases present with a pre-existing neurocognitive disorder, indicating the need for routine cognitive assessment in hospitals [17,18]. A prospective study was done to evaluate nurses’ ability to recognize delirium and its symptoms, which reveals that nurses frequently misdiagnose delirium, specifically in patients with three or further risk factors [19].

Delirium patients negatively impact their families and the healthcare system in general due to the delirium association with a drop in a person’s level of independence after a hospital discharge. A prospective study conducted in Sweden that compared the capability to perform daily life activities between delirious patients and non-delirious patients showed that delirious patients were more dependent on others than non-delirious patients [20]. Delirium patients affect the healthcare system by raising healthcare costs, especially in cases that develop delirium following elective surgery, which requires prolonged hospitalization and an increased level of care [21]. Moreover, a study done in Saudi Arabia shows that the prevalence of delirium among recently admitted hospitalized elderly patients (60 years old and above) is 21.8% (32 out of 147) [22]. Hence, the aim of the present study is to assess the knowledge, risk factors, and attitudes of the public about delirium.

## Materials and methods

This study was conducted in Saudi Arabia from May 2023 to March 2024. This study involved the participation of all residents aged 18 or older in Saudi Arabia. The sample was selected by following specific inclusion criteria, encompassing individuals from Saudi and non-Saudi residents, male and female, and expressing an interest in participating in the study throughout the Kingdom. People under 18 and people who did not agree to participate were excluded. The electronic questionnaire composed of 36 items divided into four sections [4]. The first section (questions 1-6) was focused on participants’ demographic data, the second (questions 7-16) is related to the general knowledge of delirium, the third (questions 17-32) is related to risk factors of knowledge of delirium, and the last section (questions 33-36) is related to the attitude of participants toward delirium. Since the items on the questionnaire were taken from earlier research publications and adapted, it is valid for use in this study. The questionnaires of the second and third sections (knowledge and risk factor questions, respectively) were developed, and Hare et al.'s study was used to determine nurses’ knowledge of delirium and its risk factors [23]. In addition, we adapted these questions used for assessing attitudes (fourth section) that were developed, piloted, and validated by Patel et al., which was done to assess attitudes and behaviors of healthcare professionals on delirium in ICU [24].

Data collection was conducted through a self-administered questionnaire using a standardized Nordic questionnaire format to evaluate the public’s knowledge, risk factors, and mindset. The questionnaire was distributed electronically to adult male and female participants. The sample size of 1,470 was calculated with a margin of error of 5% and a confidence level of 95% using the Raosoft sample size calculator. The collected dataset was subjected to a comprehensive statistical analysis, including descriptive and inferential methodologies. Sociodemographic characteristics and categorical variables were examined using percentages and frequencies, and the outcomes were tallied. For knowledge assessment, a scoring system was used, with correct answers assigned a value of "1" and incorrect or uncertain answers assigned "0." The scores were summed, and the participants with more than 50% correct answers were classified as having a high level of knowledge.

Statistical tests such as the Mann-Whitney U test and chi-square/Fisher's exact test were employed to evaluate the differences in attitude, knowledge about risk factors, and sociodemographic factors. A p-value of 0.05 or lower and a 95% confidence Interval were considered statistically significant. IBM SPSS Statistics for Windows, version 29.0 (released 2023, IBM Corp., Armonk, NY) was used for all statistical analyses. Ethical consent was obtained from the participants in the electronically distributed questions, who were assured of confidentiality and informed that their responses would be used solely for research purposes. The Research Ethics Committee (REC) at the University of Hail (Hail, Saudi Arabia) issued approval H-2023-318.

## Results

Our study included 1,470 participants. Table 1 shows that the majority were female (79.1%), had Saudi nationality (89.9%), and unmarried (65.4%). Age distribution showed a significant proportion in the 18-24 years' category (59.5%). Educational status varied, with 57.3% having a bachelor's degree. Geographic distribution indicated diverse residence areas within Saudi Arabia, with the largest group in the eastern area (30.8%).

**Table 1:** Distribution of the sample according to the sociodemographic variables (n = 1470) n = frequency, % = percentages

		Frequency (n)	Percent (%)
Gender	Female	1163	79.1
	Male	307	20.9
Age	18–24 years	875	59.5
	25–39 years	342	23.3
	40–60 years	231	15.7
	>60 years	22	1.5
Nationality	Non-Saudi	148	10.1
	Saudi	1322	89.9
Marital status	Married	508	34.6
	Unmarried	962	65.4
Area of residence	Central area	271	18.4
	Eastern area	453	30.8
	Northern area	216	14.7
	Southern area	172	11.7
	Western area	358	24.4
Educational status	Less than secondary education	40	2.7
	Secondary education	547	37.2
	Bachelor	843	57.3
	Masters	30	2.0
	Doctoral	10	.7

**Figure 1:**
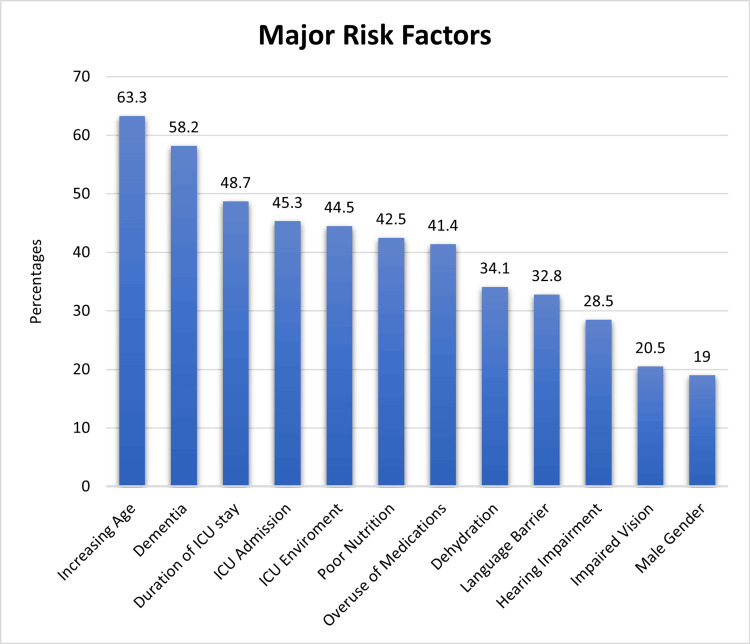
Results of questions regarding the risk factor of delirium (n = 1470)

Figure 1 shows a graph that demonstrates the major risk factors identified by the participants. The most recognized factors included increasing age (63.3%), dementia (58.2%), longer ICU stay (48.7%), ICU admission (45.3%), and ICU environment (44.5%). Other factors mentioned were poor nutrition, greater medication use, dehydration, language barrier, hearing impairment, impaired vision, and male gender, with varying percentages.

Table 2 shows the insights into general knowledge about delirium among the participants, with percentages reflecting correct answers. Notably, 29.7% recognized that symptoms of depression can mimic delirium, while 33.9% correctly disagreed that delirium lasts only a few hours. Furthermore, 33.8% acknowledged that patients with delirium have a higher mortality risk, and 63.9% correctly identified that behavioral changes throughout the day are typical of delirium. However, some correctly disagreed about the facts, such as 33.1% believing that patients with delirium are always aggressive, 15.2% thinking that patients never remember delirium episodes, 40.3% understanding that delirium involves orientation fluctuations, and 27.1% recognizing that all delirium treatment includes sedation. In addition, 74.2% recognized that delirium can lead to attention difficulties, while 58.6% understood that an altered sleep/wake cycle may be a symptom.

**Table 2:** Results of questions regarding general knowledge of delirium (n = 1470) n= frequency, % = percentages. Note: percentages reflect correct answers.

	Correct answer	Frequency (n)	Percent (%)
Symptoms of depression may mimic delirium.	True	437	29.7
Delirium never lasts for more than a few hours.	False	498	33.9
Patients with delirium have a higher risk of mortality than patients without delirium.	True	497	33.8
Patients with delirium are always physically and/or verbally aggressive.	False	487	33.1
Behavioral changes in the course of the day are typical of delirium.	True	939	63.9
Patients never remember episodes of delirium.	False	223	15.2
The fluctuation between orientation and disorientation is not typical of delirium.	False	592	40.3
Treatment for delirium always includes sedation.	False	398	27.1
A patient with delirium is likely to be easily distracted and/or have difficulty following a conversation.	True	1091	74.2
Altered sleep/wake cycle may be a symptom of delirium.	True	861	58.6

Table 3 shows knowledge about risk factors for delirium. Many participants correctly recognized that increasing aging (63.3%), the use of multiple drugs (41.4%), dementia (58.2%), impaired vision (20.5%), poor nutrition (42.5%), male gender (19.0), dehydration (34.1%), hearing impairment (28.5%), ICU length of stay (48.7%), reason for ICU admission (45.3%), ICU environment (44.5%), and language barriers (32.8%) are indeed risk factors for delirium. Notably, some participants disagreed that obesity, diabetes, a family history of dementia, and gender were risk factors, indicating potential areas for education and awareness.

**Table 3:** Results of questions regarding risk factors of delirium (n = 1470) n= frequency, % = percentages. Note: percentages reflect correct answers.

	Correct answer	Frequency (n)	Percent (%)
The risk for delirium increases with age.	True	930	63.3
The greater the number of medications a patient is taking, the greater his/her risk of delirium.	True	609	41.4
Obesity is a risk factor for delirium.	False	538	36.6
Diabetes is a strong risk factor for delirium.	False	418	28.4
A family history of dementia predisposes a patient to delirium.	False	191	13.0
Dementia is the strongest risk factor for delirium.	True	855	58.2
A patient with impaired vision is at increased risk of delirium.	True	301	20.5
Gender has no effect on the development of delirium.	False	369	25.1
Poor nutrition increases the risk of delirium.	True	625	42.5
Males are more at risk for delirium than females.	True	280	19.0
Dehydration can be a risk factor for delirium.	True	502	34.1
Hearing impairment increases the risk of delirium.	True	419	28.5
Do you think the length of ICU stay plays a role in delirium?	True	716	48.7
Do you think the reason for ICU admission plays a role in delirium?	True	666	45.3
Do you think ICU environment style plays a role in delirium?	True	654	44.5
Do you think language barriers between patients and nurses play a role in delirium?	True	482	32.8

Table 4 reflects the attitudes of participants toward delirium management and outcomes. The responses are categorized from "Totally disagree" to "Totally agree" on various statements. A significant proportion (30.7%) agreed and totally agreed that delirium requires active intervention, while 17.1% considered it largely preventable. A notable number (17.5%) felt that delirium is underdiagnosed, and 16.5% saw a connection between delirium and oversedation in the ICU.

**Table 4:** Questions related to the attitude of participants toward delirium management and outcomes (n = 1470) n = frequency, % = percentages

		Totally disagree	Disagree	Neutral	Agree	Totally agree
Delirium is a problem that requires active intervention.	N	653	82	269	272	180
	%	44.4	5.6	18.3	18.5	12.2
Delirium is largely preventable.	N	615	95	493	153	99
	%	41.8	6.5	33.5	10.4	6.7
Delirium is an under-diagnosed syndrome.	N	386	243	557	100	157
	%	26.3	16.5	37.9	6.8	10.7
Delirium is related to oversedation in the ICU.	N	425	205	582	99	143
	%	28.9	13.9	39.6	6.7	9.7

Table 5 analyzes the association between attitudes toward delirium management and knowledge of its risk factors. No significant differences in attitudes were found between the two knowledge groups, suggesting that knowledge of delirium risk factors does not strongly influence attitudes toward delirium management.

**Table 5:** Sociodemographic characteristics and general knowledge about delirium (n = 1470)

			General knowledge about delirium		Sig. value
			Lower knowledge	Higher knowledge	
Gender	Female	N	605	558	0.001
		%	76.0%	82.8%	
	Male	N	191	116	
		%	24.0%	17.2%	
Age	18–24 years	N	443	432	0.003
		%	55.7%	64.1%	
	25–39 years	N	211	131	
		%	26.5%	19.4%	
	40–60 years	N	127	104	
		%	16.0%	15.4%	
	>60 years	N	15	7	
		%	1.9%	1.0%	<0.001
Marital status	Married	N	316	192	
		%	39.7%	28.5%	
	Unmarried	N	480	482	
		%	60.3%	71.5%	
Nationality	Non-Saudi	N	61	87	<0.001
		%	7.7%	12.9%	
	Saudi	N	735	587	
		%	92.3%	87.1%	
Place of residence	Central area	N	155	116	0.073
		%	19.5%	17.2%	
	Eastern area	N	242	211	
		%	30.4%	31.3%	
	Northern area	N	117	99	
		%	14.7%	14.7%	
	Southern area	N	106	66	
		%	13.3%	9.8%	
	Western area	N	176	182	
		%	22.1%	27.0%	
Educational level	Less than secondary education	N	21	19	0.299
		%	2.6%	2.8%	
	Secondary education	N	285	262	
		%	35.8%	38.9%	
	Bachelor	N	468	375	
		%	58.8%	55.6%	
	Masters	N	19	11	
		%	2.4%	1.6%	
	Doctoral	N	3	7	
		%	0.4%	1.0%	

Table 6 shows the association between sociodemographic factors and general knowledge of delirium. There are significant associations between several sociodemographic variables and knowledge levels. Notably, females and younger age groups (18-24 and 25-39 years) showed higher knowledge levels (p = 0.001, 0.003). Unmarried individuals and Saudi nationals also exhibited better knowledge (p < 0.001). Age, gender, marital status, and nationality were influential factors in participants' general knowledge about delirium, shedding light on disparities in awareness within the population.

**Table 6:** Associations of attitude toward the management and outcomes of delirium with knowledge about risk factors of delirium (n = 1470) Note: The lower mean shows a negative attitude while the higher mean shows a positive attitude based on a five-point Likert scale.

	Knowledge about risk factors of delirium				Sig. value
	Poor knowledge		Good knowledge		
	N	Mean (SD)	N	Mean (SD)	
Delirium requires active interventions.	948	3.51 (1.15)	508	3.53 (1.32)	0.099
Delirium is preventable.	949	3.45 (0.87)	506	3.40 (1.19)	0.117
Delirium is an underdiagnosed syndrome.	947	3.05 (0.96)	496	2.96 (1.24)	0.238
Delirium is related to sedative overuse in the ICU.	948	3.10 (0.92)	506	3.08 (1.24)	0.393

Table 7 shows the sociodemographic factors related to patients' attitudes toward delirium management. Gender influences attitudes significantly, with females having a more positive attitude (p = 0.001). Age impacts attitudes, with younger participants being more positive (p < 0.001). Marital status (p < 0.001), nationality (p = 0.017), and geographic location (p = 0.011) also affect attitudes. Educational level did not show significant differences in terms of attitude towards delirium.

**Table 7:** Sociodemographic characteristics and attitude of patients toward the management and outcomes of delirium (n = 1470)

			Attitude towards delirium		Sig. value
			Negative attitude	Positive attitude	
Gender	Female	N	451	701	0.001
		%	74.1%	82.5%	
	Male	N	158	149	
		%	25.9%	17.5%	
Age	18–24 years	N	321	544	<0.001
		%	52.7%	64.0%	
	25–39 years	N	185	157	
		%	30.4%	18.5%	
	40–60 years	N	89	141	
		%	14.6%	16.6%	
	>60 years	N	14	8	
		%	2.3%	0.9%	<0.001
Marital status	Married	N	262	244	
		%	43.0%	28.7%	
	Unmarried	N	347	606	
		%	57.0%	71.3%	
Nationality	Non-Saudi	N	47	98	0.017
		%	7.7%	11.5%	
	Saudi	N	562	752	
		%	92.3%	88.5%	
Place of residence	Central area	N	121	148	0.011
		%	19.9%	17.4%	
	Eastern area	N	163	287	
		%	26.8%	33.8%	
	Northern area	N	83	130	
		%	13.6%	15.3%	
	Southern area	N	86	86	
		%	14.1%	10.1%	
	Western area	N	156	199	
		%	25.6%	23.4%	
Educational level	Less than secondary education	N	17	22	0.536
		%	2.8%	2.6%	
	Secondary education	N	230	311	
		%	37.8%	36.6%	
	Bachelor	N	343	497	
		%	56.3%	58.5%	
	Masters	N	16	13	
		%	2.6%	1.5%	
	Doctoral	N	3	7	
		%	0.5%	0.8%	

## Discussion

Delirium is a transient state of confusion characterized by cognitive dysfunction. Various factors can contribute to delirium, including medical conditions, severe neuropsychiatric disorders such as depression and dementia, the elderly population, substance abuse, medication, hospitalization in the ICU, or a combination of these. It disproportionately affects older individuals and can lead to adverse outcomes like increased mortality and healthcare costs. Our study provides valuable insights into the awareness and perceptions of delirium among the general population in KSA and explores risk factors. Our discussion will focus on the primary findings and their implications, shedding light on the significance of the study's outcomes.

In our study, we noted that the majority of participants were young, unmarried, Saudi, female, and well-educated. This demographic profile allows us to get important information about delirium awareness and attitudes among the diverse and wide portion of Saudi Arabian society with a special focus on a younger and more varied cohort. This information may help identify if the young group of the general population needs focused educational interventions.

A study conducted in Wisconsin, USA, shows that family caregivers struggled to distinguish delirium symptoms from dementia symptoms. It was also more difficult to identify delirium symptoms [25]. According to another study conducted in Saudi Arabia, knowledge of delirium and its risk factors is insufficient [4]. Likewise, in this study, only three out of 10 questions assessing general knowledge of delirium were correctly answered by more than 50% of the participants. According to the study results, more than half of the participants (66.1%) believed incorrectly that delirium only lasts a few hours, and just 33.9% of people accurately disagreed with this assertion. Given the well-established detrimental effects of prolonged episodes of delirium, as reported by Bellelli et al. (2007) and González et al. (2009), this misperception regarding the length of delirium is troubling. As a result, the health and well-being of older persons may suffer significantly if delirium symptoms are not identified early enough and proper treatment is not sought. For elderly people suffering from delirium, it is imperative to correct this misconception and raise awareness among the public and medical professionals to guarantee prompt response and maximize outcomes [26,27].

One of the findings indicates that knowledge of delirium risk factors does not significantly affect attitudes toward its management. This contrasts with previous research, which has shown that education about medical conditions (through e-learning programs) often leads to more positive attitudes and improved management [28]. It suggests that in the context of delirium, factors beyond knowledge may play a stronger role in shaping attitudes. Further studies could explore these factors to develop more effective strategies for improving delirium management attitudes in the general public.

A cross-sectional study about delirium knowledge, risk factors, and attitude was conducted by the general public in Riyadh, Saudi Arabia, shows insufficient knowledge in the general public about delirium and its risk factors [4].

In our study, the participants' knowledge of risk factors associated with delirium, such as increasing age, dementia, and ICU-related factors, was good. There were some false beliefs, such as some participants believing that obesity, diabetes, and a family history of dementia were risk factors for delirium. Targeted education has the potential to dispel these myths and improve public knowledge of the risk factors for delirium.

In this study, we explored the attitudes of participants toward delirium and examined the associations between these attitudes and their knowledge about the risk factors of delirium. A cross-sectional study was conducted in Riyadh, Saudi Arabia, to assess the knowledge of delirium, its risk factors, and attitude toward it [4]. The findings of this study suggest that a significant proportion of participants recognize delirium as a problem requiring active intervention and perceive it as largely preventable. Notably, a considerable percentage of respondents also associate delirium with over-sedation in the intensive care unit. Regarding our findings, the responses revealed diverse perspectives on delirium. There is a significant number of people who believe that delirium requires active intervention, while others see it as preventable. Moreover, some participants believed that delirium is underdiagnosed, and it was acknowledged that there could be a connection between delirium and oversedation in the ICU. The complexity of perceptions surrounding delirium is demonstrated by these findings, which suggest areas for further investigation and intervention.

Sociodemographic factors significantly influence attitudes toward delirium management. Age emerges as a significant factor influencing awareness or knowledge [29]. Moreover, most participants, particularly those in the 18-39 age group, demonstrated a more positive attitude toward delirium management [4]. Our findings confirm that age influences attitudes, as younger participants are more likely to be positive (p < 0.001). Furthermore, the age groups of 18-24 and 25-39 years displayed higher knowledge levels (p = 0.001, 0.003). This highlights the importance of considering age as a significant factor in understanding attitudes toward delirium management and knowledge about the condition. They may be more willing to participate in health education programs related to delirium. However, it is essential to continue educating individuals across all age groups to ensure a comprehensive understanding of delirium. Other factors influence attitudes toward delirium; for example, geographic location within Saudi Arabia (p = 0.011) also plays a role in shaping attitudes. Residents of the eastern area showed a significantly more positive attitude toward delirium. This regional variation underscores the importance of tailoring educational initiatives to address the specific needs and awareness levels of different geographic regions. Notably, gender was found to have a significant impact, with females exhibiting a more positive attitude toward delirium (p = 0.001). Marital status (p < 0.001) and nationality (p = 0.017) also played significant roles in shaping attitudes. This aligns with the previous literature in which marital status shows a significant impact on the attitude toward delirium [30]. However, the educational level did not show any significant difference in attitude toward delirium. This contrasts with some prior studies that have shown significant educational disparities in healthcare perceptions with more positive attitudes toward higher education [2]. It suggests the need for targeted educational interventions to bridge this gap in understanding delirium management.

In our study, there are some limitations, such as the number of participants and the questionnaire could have more specific information about the source of the participants’ knowledge, which help us to correct the misconceptions by implementing the best educational strategies. Based on the information we noted in these results, delirium education programs should be considered for the general public to improve knowledge of delirium.

## Conclusions

This study assessed the delirium knowledge and risk factors among the population of Saudi Arabia. We found a generally good understanding of key risk factors, such as increasing age, dementia, and ICU-related factors. However, we found some misconceptions. For example, some participants mistakenly believed that obesity, diabetes, and a family history of dementia were risk factors for delirium. We recommend future researchers to conduct surveys manually instead of online for more accurate results. Furthermore, the Saudi population needs more attention through seminars, campaigns, and media commercials about delirium.
